# A Review of the Cost Effectiveness of Transcatheter Aortic Valve Replacement (TAVR) Versus Surgical Aortic Valve Replacement (SAVR)

**DOI:** 10.7759/cureus.46535

**Published:** 2023-10-05

**Authors:** Jonathan Kermanshahchi, Birpartap Thind, Gabriel Davoodpour, Megan Hirsch, Jeff Chen, Akshay J Reddy, Evan Chan, Zeyu YU, Daryoush Javidi

**Affiliations:** 1 Medicine, California University of Science and Medicine, Colton, USA; 2 Medicine, California Northstate University, Elk Grove, USA; 3 Medicine, California Health Science University, Clovis, USA; 4 Medical Education, California University of Science and Medicine, Colton, USA

**Keywords:** savr, tavr, cost, aortic valve, tavr vs savr, cost of savr, cost of tavr, surgical aortic valve replacement, transcatheter aortic valve replacement

## Abstract

The cost of transcatheter aortic valve replacement (TAVR) has been studied in the context of high-risk or specific comorbidity populations; this paper provides a comprehensive overview of broader patient populations’ outcomes and costs with TAVR in comparison to surgical aortic valve replacement (SAVR). In the past, SAVR had been the more cost-effective option than TAVR, but in recent years, TAVR has been becoming more cost-effective.Though the cost of TAVR can vary due to several factors the major focus of this review will focus on the surgical technique, medicare reimbursements, insertion point, and varying risk populations. In conclusion, the price of TAVR is declining as more cost-efficient valves arrive on the market. Climbing healthcare costs play a significant role in clinical decisions when deciding on which procedures are most cost-effective for the patient and healthcare system. The declining price of TAVR could lead to the preference of TAVR over SAVR for both low-risk and high-risk aortic stenosis patients.

## Introduction and background

Cardiovascular diseases remain a significant health burden, and valvular heart disease is a prevalent condition among the aging population. Among the various ways to treat aortic valve conditions, transcatheter aortic valve replacement (TAVR) and surgical aortic valve replacement (SAVR) stand at the forefront of therapeutic interventions [1]. The factors that go into deciding which therapeutic intervention will best benefit patients are multifactorial, with cost being a major factor in the decision as healthcare moves toward the pursuit of value-based medicine. Literature has shown that the TAVR device cost makes up the bulk of the cost, ultimately bringing up the price for the procedure compared to SAVR [1]. In 2011, TAVR was introduced to clinical practice for patients at high risk of undergoing open heart surgery. Around this time, the device cost was at a relatively high point, which questioned its cost-effectiveness when compared to its surgical counterpart SAVR. Recent trends have shown a decrease in valve prices and overall procedure costs as more patients are able to receive this procedure because of its approval for low-risk patients. Given the increased patient population who are able to receive TAVR, it is crucial to include economic considerations on top of clinical indications in order to rule out which patients would benefit from undergoing TAVR versus SAVR. This review article includes low-to-high-risk patient populations undergoing TAVR procedures.

## Review

Methods

**Figure 1:**
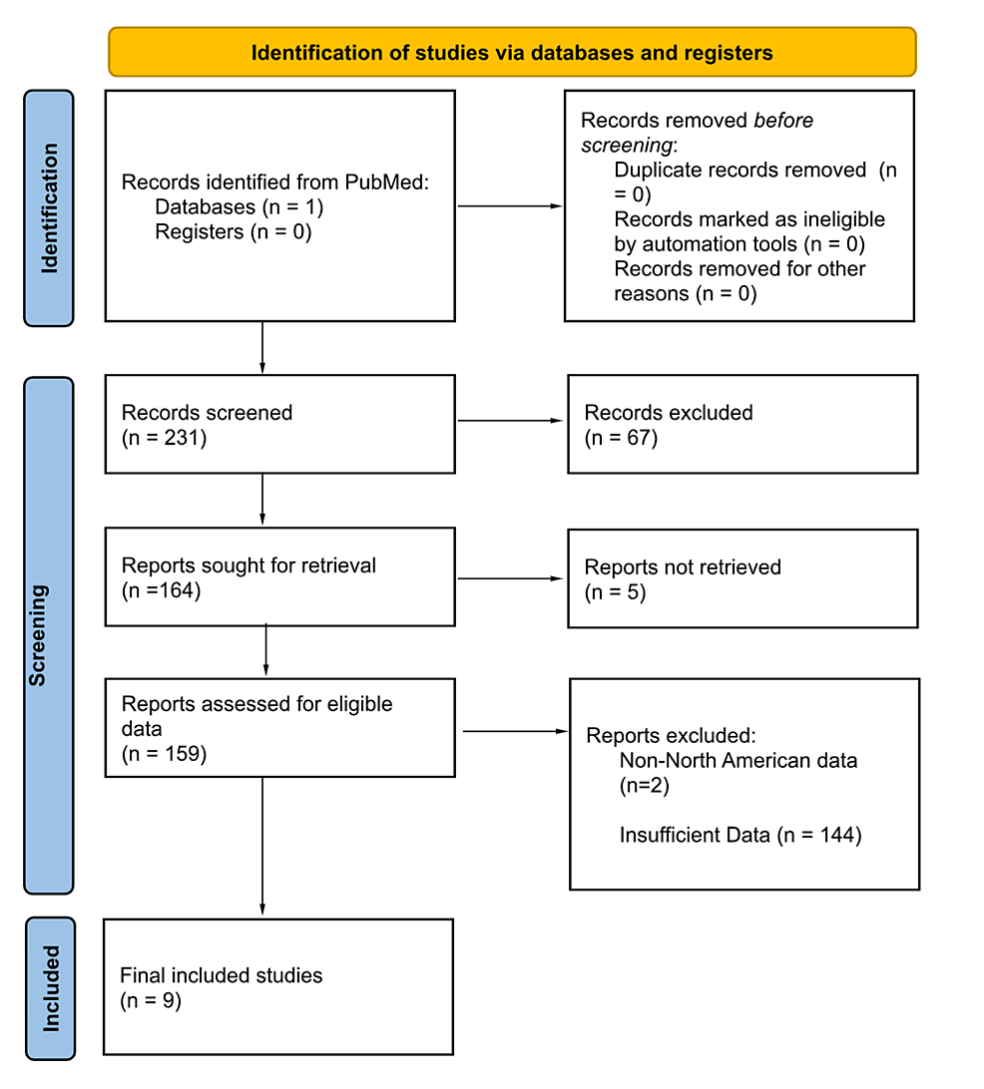
PRISMA Diagram for Reviewed Studies PRISMA: Preferred Reporting Items for Systematic Reviews and Meta-Analyses

For this comprehensive study, we aimed to investigate the cost-effectiveness of TAVR compared to SAVR in aortic stenosis patients, encompassing broader patient populations. The analysis also considered the impact of varying comorbidities on costs. We conducted a systematic literature search from September 2022 to November 2022 using the primary search engine, Pubmed, to identify relevant publications in the field. The search term "TAVR and costs" yielded a total of 231 publications from the past 10 years. After thorough screening, we included studies that met specific criteria, including North American studies, those with patient data, studies explicitly comparing TAVR and SAVR, and those discussing the cost of TAVR and its trends over time. Our focus encompassed all risk groups and various procedural methods with different types of valves used. The selected articles were reviewed to assess the evolving cost dynamics of TAVR compared to SAVR. The analysis mainly concentrated on the impact of surgical technique, medicare reimbursements, insertion points, and diverse risk populations on the cost differences between TAVR and SAVR. To draw meaningful conclusions, we carefully analyzed the relevant literature, considering the variations in TAVR costs influenced by multiple factors. The results for this search and filtration are displayed in Figure 1. It is to be noted that the dollar amounts listed throughout the paper have not been altered except for the two articles [1,2] as they have been converted from Canadian dollars from the respective years mentioned in the studies to 2022 U.S.D.

Valve variations, techniques, and insertion points

Once a patient is matched to be a good candidate for the procedure, dependent upon risk factors, next is the execution of the procedure. The execution can be performed in several variations including but not limited to, using femoral artery, transapical access, transcaval access, transcarotid access, and subclavian access.

Of the options listed, transfemoral access and trans axillary are the most common. Transfemoral is the preferred method for TAVR, given its minimally invasive nature and the fact that it can be performed under conscious sedation without intubation, further limiting complications that may arise [3]. In fact, a study showed there was a 20% relative reduction in mortality when comparing transfemoral TAVR (TF-TAVR) with SAVR (hazard ratio (HR), 0.80; 95% CI, 0.69-0.93; P=0.024) [4]. Some of the most common complications for this technique are bleeding and or hematoma at the site of access [5].

The second most common option after transfemoral is the trans axillary approach which is a viable option in 5-10% of patients [3]. However, it should be noted that this technique has some caveats that can increase complications and thus costs associated with the procedure such as stroke [6]. The right axillary or subclavian artery has challenging anatomy due to an unfavorable angle for TAVR implantation. As such the proximal third of the left axillary artery is a better candidate for this procedure [6]. All in all, if these two sites are inaccessible, physicians may use transaortic access, do transcaval (especially in high-risk populations), or use transcarotid access.

Although major differences lie within TAVR vs. SAVR, it is important to not forget that there are intraprocedural differences when using TAVR that affect outcome and cost. Valves utilized in TAVR are a large portion of the overall cost, and it is crucial to understand the benefits as well as drawbacks of using various types of valves. The two most commonly used valves in TAVR are the self-expanding valves (SEV) and the balloon-expandable valves (BEV) [7]. Even though there are large differences between the two valves, they both can be used interchangeably [7]. BEV favors the wing structures of the stent which surrounds it as it expands and anchors the stent into place [7]. SEV is both repositionable and retrievable when in the final position [8].

A large randomized study focused on comparing the two types of valves looked at the occurrence of paravalvular regurgitation (PVR), which is associated with long-term mortality risk [9]. The results of this study demonstrated that SEV was associated with a higher risk of PVR [9]. In addition, SEV demonstrated a higher in-hospital and two-year mortality compared to BEV [9].

Another study compared outcomes of the SEV TAVR procedures with SAVR in high-risk patients, similar mid-term survival and stroke rates were found following the interventions [10]. In addition, valve reinterventions and deterioration were uncommon after the procedure [10].

The total cost for TAVR on average has been higher (index procedure + nonprocedural + physician fees) at $69,592 ± $24,387 compared to SAVR’s total cost of $58,332 ± $32,653; though TAVR excels and may offset cost due to shorter hospital stay and post-discharge care, it is still not enough to provide a cost-benefit for TAVR [11]. However, the authors mentioned that if the length of stay is at least five to six days shorter with TAVR than SAVR, cost neutrality could be achieved [11]. Another study looking at the cost-effectiveness of TAVR replacement in patients with severe aortic stenosis in intermediate-risk populations using the PARTNER 2 (Placement of Aortic Transcatheter Valves 2) trial discovered that procedural costs were approximately $20,000 higher with TAVR than SAVR [12]. When breaking the cost by types of valve, the total cost differences for the index hospitalization were $2,888 higher with Sapien XT valve TAVR (Edwards Lifesciences, CA), but were $4,155 lower with Sapien 3 valve TAVR (Edwards Lifesciences, CA) [12]. Furthermore, Baron and colleagues found that TAVR demonstrated a projected lifetime value lower cost by $8,000-10,000 while also increasing quality-adjusted survival by 0.15 to 0.27 years relative to SAVR [12].

Over a shorter time horizon (six-month total inpatient healthcare expenditures), Goldsweig et al. found costs of $64,395 for TAVR and $59,743 for SAVR [13]. This study also measured the procedural cost for TA-TAVR (an approach typically reserved for patients who have an unfavorable transfemoral approach); likewise, the cost for TA-TAVR remained higher than SAVR [13]. However, the follow-up cost for TAVR differed depending on the access site, with the estimated mean cost of follow-up hospitalizations was $18,122±58 142 (TF-TAVR) and $11,733±31 924 (transapical TAVR (TA-TAVR)) using the SAPIEN heart valve system; though TA-TAVR is associated with an increased risk of 30-day readmission, influenced by residual confounding as patients undergoing TA-TAVR are typically sicker [14]. Other valves that showed lower follow-up cost for TAVR were XT-TAVR (Δ=-$9304) and S3-TAVR (Δ=-$11 377) compared with SAVR [12]. Furthermore, the price of a one-year follow-up for all risk groups was $9,763 for TAVR recipients and $14,073 for SAVR recipients due to the lower likelihood of postoperative complications [12]. The lower cost trend continued when looking at the mean readmission (TAVR: $3,548 ± $10,669; SAVR: $4,698 ± $14,781) and post-acute care payments (TAVR: $6,229 ± $9,020; SAVR: $6,968 ± $9,066) [15].

There are also variations in SAVR, such as valve types and entry points, that affect the costs [16]. A recent study in 2022 investigated the new aortic valve surgical technique, sutureless aortic valve replacement (SuAVR), and compared the costs associated with this procedure to TAVR [16]. The study interestingly found that even though TAVR had better postoperative outcomes which included fewer incidents necessitating blood transfusions, as well as a shorter length of stay, costs were less in SuAVR [16]. The study mainly attributes this to the high costs of the implanted prosthesis [16]. It is important to note that the cost analysis for this study was carried out in a health system that is not generalizable to all countries that have a system linked to health insurance [16]. However, the authors believe that since the cost was mainly attributed to the cost of the devices used, countries with different healthcare systems would obtain similar results [16].

Risk populations

Tam DY and colleagues (2018) looked at the lifetime costs of using different valves on differing risk populations on two separate occasions [1]. In 2018 they constructed a fully probabilistic Markov model with cycle lengths of 30 days to estimate the cost and benefits of TAVR compared with SAVR over the lifetime horizon [1]. The analysis compared the self-expandable valve TAVR to SAVR, using findings from the Surgical Replacement and Transcatheter Aortic Valve Implantation (SURTAVI) trial and Canadian cost data [1]. All costs and outcomes mentioned in the study were discounted at 1.5% per annum as per guidelines from the Canadian Agency for Drugs and Technologies in Health. Taking all these factors into account the total lifetime costs (mean ± standard deviation) were $38,839.70 ± 6,426.31 for TAVR and $28,927.90 ± 11,891.45 for SAVR [1]. Tam DY and colleagues did a similar study in 2021 looking at low-surgical-risk populations. For this population, the lifetime cost was $31,156.99 ± 3,942.56 and $33,099.51 ±4,057.73, for balloon-expandable TAVR and self-expandable-TAVR, respectively [2]. Comparatively, it was $28,862.33 ± 5,617.57 for SAVR arms [2]. TAVR’s lifetime costs here are shown to be more than SAVR’s contrary to a study by Baron and colleagues [1,2,12].

Because TAVR is being expanded to a broader population, it is important to understand how other risk factors will affect treatment. One study found that anatomical variations can increase the risk of developing a bundle branch block following TAVR, including the His bundle penetrating the left side or under the membranous septum [17]. Additionally, preexisting bundle branch blocks are the most common reason for permanent pacemaker implantation [17]. Patients with bicuspid valves are known to develop premature aortic stenosis and regurgitation, and this anatomical difference has been found to make anchoring the TAVR device much more difficult during the procedure [18]. This difficulty is not associated with higher mortality, but it is associated with higher procedural complication rates [18]. Another study states that younger patients (less than 65 years of age) will likely need a new valve later in life and should get SAVR instead of TAVR [19]. Additionally, this paper states that people with rheumatic heart disease, any severe calcifications, aortopathy, or severe septal hypertrophy should not be candidates for TAVR [19]. One paper created a risk prediction method, identifying gait speed, grip strength, body mass index, anemia, hypoalbuminemia, unexpected weight loss, and impaired mobility on the basis of recent falls or being wheelchair-bound as key variables for determining risk for TAVR [20]. Finally, patients with pure aortic regurgitation may benefit from TAVR over SAVR with new devices on the horizon, but it has also been found that due to calcifications, these patients suffer worse outcomes, even when using new-generation valves [21].

Medicare cost coverage

While discussing the costs for the procedures it is important to note that 91.6% of TAVR patients discharged alive relied on Medicare as their primary payer so it is important to consider the cost for Medicare beneficiaries [14]. TAVR has constantly remained as solely reimbursed by Medicare for the most part and since the procedure began being used in 2012 medicare reimbursement rates have consistently fallen over the years based on a 2022 study [22]. TAVR hospitalizations increased from 6,865 in 2012 to 17,925 in 2014 among those under Medicare and as a result, overall medicare spending has also increased from $0.4 billion in 2012 to $1 billion in 2014 for the TAVR procedure [23]. Medicare pays for TAVR across all risk groups, with the cost of TAVR index hospitalization being $61,845 for low-risk surgical, $64,658 for intermediate risk, and $65,694 for high-risk [12]. Brescia further breaks down the costs and compares it to SAVR [15]. They found that 90-day episode payments were $69,388 ± $22,259 for TAVR versus $66,683 ± $27,377 for SAVR [15]. Furthermore, mean index hospitalization payments were higher for TAVR being $51,472 ± $9,430 versus $47,098 ± $16,005 for SAVR [15]. However, mean readmission (TAVR: $3,548 ± $10,669; SAVR: $4,698 ± $14,781) and post-acute care payments (TAVR: $6,229 ± $9,020; SAVR: $6,968 ± $9,066) were lower for TAVR compared to SAVR [15]. A 2015 study using a propensity-matched sample found that medicare payments for TAVR hospitalizations at $52,200 ± $28,200 were lower when compared with SAVR hospitalizations at $56,300 ± $32,600 [24]. However, this same study found that SAVR hospitalizations represented a net profit for providers but a net loss for TAVR hospitalizations [24]. Another 2017 comparative study found that medicare payments were statistically significantly lower for TAVR hospitalizations (median, $49,500) than for SAVR hospitalizations (median, $50,400) [25]. Furthermore, hospital costs were higher for TAVR compared to propensity-matched SAVR patients (TAVR: median, $50,200; SAVR: median, $45,500) [26]. Again, the increased hospital costs were mainly attributed to the higher medical supply costs that come with TAVR, such as the implanted valve prosthesis [14-16,26]. Similarly, a study from 2019 looked at Medicare episodic payments for TAVR and SAVR, 90 days before aortic valve replacement through 90 days after hospital discharge (2012-2015) [25]. Overall, patients who underwent TAVR had an average of $3922 lower-adjusted total episode payments than those who underwent SAVR (difference $3922, [95% CI, $2,731-5,113]; P<0.001) [25]. TAVR was associated with $1399 higher-adjusted preprocedural payments (difference $1,399 [95% CI, $1,124-1,675]; P<0.001), but $1560 lower-adjusted payments for the index hospitalization (difference, $1,560 [95% CI, $791-2,328]; P<0.001) and $3733 lower-adjusted payments in the postprocedure period (difference, $3,733 [95% CI, $3,012-4,454]) [25]. Thus showing that Medicare payments typically tend to be lower for TAVR compared to SAVR [25].

Furthermore, different hospitals may not have the same outcomes for the TAVR procedure among medicare beneficiaries which can lead to alterations in the overall cost of the procedures. A 2015 study using data from all Medicare fee-for-service beneficiaries greater than 65 years of age between the years of 2011 and 2013 investigated hospital variation of TAVR based on risk-standardized 30-day mortality, one-year mortality, and 30-day all-cause readmission [27]. Hospitals were measured based on their average 30-day mortality rate [27]. Overall, there was a two-fold increase in the odds ratio of death at a hospital one standard deviation above the national average compared to hospitals one standard deviation below the national average [27].

The trend

**Figure 2:**
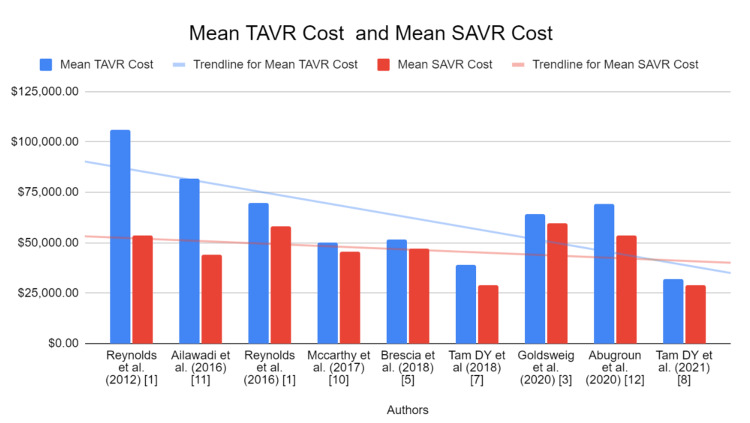
Mean TAVR/SAVR Cost for Various Studies The general trend from 2012 to 2021 shows a decrease in the costs for both SAVR and TAVR. However, it is the cost of TAVR that has been on a steeper decline, almost matching SAVR in 2021. TAVR: transcatheter aortic valve replacement, SAVR: surgical aortic valve replacement

It is to be noted that this high cost of TAVR is mainly attributed to the cost of the valve itself, as can be seen above with varying valve types [28]. Figure 2 shows how the cost for TAVR has been trending down [1,2,11-16,25,26,28-30]. Ten years ago, TAVR was found to have a significantly higher one-year cumulative cost than SAVR, with a median of $106,076 compared to SAVR’s $53,621 [11]. As Table 1 demonstrates, just four years later, Ailawadi and colleagues’ study showed that TAVR was found to have a mean total cost of $81,638 compared to SAVR’s mean total cost of $43,974 [28]. In 2020, the gap between TAVR and SAVR’s total costs aligned with the previous trend and continued to decrease, with TAVR’s median cost (25th to 75th IQR) between $52,571 and $85,899 and SAVR’s median cost (25th to 75th IQR) between $38,461 and $68,592 [29]. Similar downward trends were found by Kawsara and colleagues as it was determined that the median cost of TAVR hospitalizations has decreased from $56,022 to $46,101 over time [30]. This is in contrast to 2012 data analyzed by Reynold and colleagues who have found a larger difference in the price between TAVR and SAVR [31].

**Table 1:** TAVR vs. SAVR Cost Comparison TAVR: transcatheter aortic valve replacement, SAVR: surgical aortic valve replacement

Authors	TAVR Cost (Mean ± S.D.)	SAVR Cost (Mean ± S.D.)	Notes
Tam DY et al. (2021) [2]	$31,156.99 ± 3,942.56	$28,862.33 ± 5,617.57	Balloon-expandable
Abugroun et al. (2020) [29]	$52,571-$85,899	$38,461-$68,592	Median cost (25th to 75th IQR)
Goldsweig et al. (2020) [13]	$64,395	$59,743	N/A
Tam DY et al. (2018) [1]	$38,839.70 ± 6,426.31	$28,927.90 ± 11,891.45	N/A
Brescia et al. (2018) [15]	$51,472 ± $9,430	$47,098 ± $16,005	N/A
Mccarthy et al. (2017) [26]	$50,200	$45,500	N/A
Reynolds et al. (2016) [11]	$69,592 ± $24,387	$58,332 ± $32,653	Index procedure + nonprocedural + physician fees
Ailawadi et al. (2016) [28]	$81,638	$43,974	N/A
Reynolds et al. (2012) [31]	$106,076	$53,621	N/A

Limitations

This study's limitations stem from its exclusive reliance on data from PubMed, potentially limiting the data pool and overlooking relevant studies from other sources. The heterogeneity of patient populations, including various risk groups and procedural methods, may introduce confounding variables, making direct comparisons between TAVR and SAVR challenging. The lack of standardized cost reporting across studies could affect the accuracy of cost comparisons. Additionally, the short timeframe of the literature search and the exclusion of non-English studies might overlook recent and global insights. The absence of long-term follow-up data and the exclusion of non-published data may introduce bias and limit the study's comprehensive conclusions. Furthermore, the study does not provide a detailed breakdown of costs and may not fully consider healthcare system variations or changing market dynamics impacting TAVR costs over time. Despite its valuable findings, researchers should address these limitations in future studies to enhance the understanding of TAVR and SAVR cost-effectiveness.

## Conclusions

Overall, the price of TAVR is declining as advancements in medical technology bring more cost-efficient valves to the market. Although SAVR still holds a strong place in treating severe aortic stenosis, the associated costs of TAVR have demonstrated a notable and encouraging downward trajectory which allows more widespread use of the procedure. However, it should be noted that the cost of TAVR is dictated by several factors, as every patient presentation is unique. A large majority of TAVR cases are reimbursed through Medicare, and Medicare spending on the procedure has greatly increased as the number of cases has increased. Clinical decisions regarding access sites and specific valve choices may affect the cost of TAVR and therefore create variability in overall costs associated with the procedure. Nonetheless, TAVR has shown promising results of being superior to SAVR not only clinically, but also financially. Further studies should be conducted in order to continue monitoring the cost of TAVR and SAVR, specifically with various comorbidities in order to validate the trend pointing toward TAVR becoming more cost-efficient than SAVR.
